# Fish Oil and the Pan-PPAR Agonist Tetradecylthioacetic Acid Affect the Amino Acid and Carnitine Metabolism in Rats

**DOI:** 10.1371/journal.pone.0066926

**Published:** 2013-06-24

**Authors:** Bodil Bjørndal, Trond Brattelid, Elin Strand, Natalya Filipchuk Vigerust, Gard Frodahl Tveitevåg Svingen, Asbjørn Svardal, Ottar Nygård, Rolf Kristian Berge

**Affiliations:** 1 Department of Clinical Science, University of Bergen, Bergen, Norway; 2 NIFES, National Institute of Nutrition and Seafood Research, Bergen, Norway; 3 Department of Heart Disease, Haukeland University Hospital, Bergen, Norway; Max Delbrueck Center for Molecular Medicine, Germany

## Abstract

Peroxisome proliferator-activated receptors (PPARs) are important in the regulation of lipid and glucose metabolism. Recent studies have shown that PPARα-activation by WY 14,643 regulates the metabolism of amino acids. We investigated the effect of PPAR activation on plasma amino acid levels using two PPARα activators with different ligand binding properties, tetradecylthioacetic acid (TTA) and fish oil, where the pan-PPAR agonist TTA is a more potent ligand than omega-3 polyunsaturated fatty acids. In addition, plasma L-carnitine esters were investigated to reflect cellular fatty acid catabolism. Male Wistar rats (*Rattus norvegicus*) were fed a high-fat (25% w/w) diet including TTA (0.375%, w/w), fish oil (10%, w/w) or a combination of both. The rats were fed for 50 weeks, and although TTA and fish oil had hypotriglyceridemic effects in these animals, only TTA lowered the body weight gain compared to high fat control animals. Distinct dietary effects of fish oil and TTA were observed on plasma amino acid composition. Administration of TTA led to increased plasma levels of the majority of amino acids, except arginine and lysine, which were reduced. Fish oil however, increased plasma levels of only a few amino acids, and the combination showed an intermediate or TTA-dominated effect. On the other hand, TTA and fish oil additively reduced plasma levels of the L-carnitine precursor γ-butyrobetaine, as well as the carnitine esters acetylcarnitine, propionylcarnitine, valeryl/isovalerylcarnitine, and octanoylcarnitine. These data suggest that while both fish oil and TTA affect lipid metabolism, strong PPARα activation is required to obtain effects on amino acid plasma levels. TTA and fish oil may influence amino acid metabolism through different metabolic mechanisms.

## Introduction

Peroxisome proliferator-activated receptors (PPARs) are ligand–activated nuclear receptors and transcription factors important for the regulation of energy metabolism, lipid storage and inflammation [1], [2]. The subtype PPARα is expressed mainly in liver and muscle and its activation is involved in the starving response, enhancing fatty acid β-oxidation and inhibiting glucose utilization [3]. Recent results demonstrate that PPARα also has an important role in the regulation of amino acid metabolism [4], [5]. In this study we wished to investigate the impact of long-term treatment with known PPARα agonists on lipid- and amino acid metabolism.

Naturally occurring PPAR ligands include fatty acids and their eicosanoid derivates, especially polyunsaturated (PUFA) fatty acids [6], [7]. Feeding studies have previously shown increased expression of PPARα response genes with fish oil (FO), in particular with high n-3 PUFA doses [8], [9]. PPAR synthetic ligands, including fibric acid derivatives and the artificial, non-β-oxidizable 3-thia fatty acid tetradecylthioacetic acid (TTA), have lipid lowering and hypoglycemic effects in rodent models [10], [11], [12]. TTA is unspecific and able to activate all subtypes of PPARs [13], [14], but its effect in rats is predominantly due to PPARα activation [11]. In humans, fibrates and omega-3 (n−3) PUFAs are utilized in the treatment of dyslipidemia, being particularly effective in reducing circulating levels of triacylglycerol (TAG) [15], [16]. However, the TAG-lowering treatment approach in cardiovascular disease is still controversial [17], [18], [19]. Thus, further studies to explore whether PPAR agonists have effects that counteract the benefits of a lowered blood lipid level are important.

**Figure 1:**
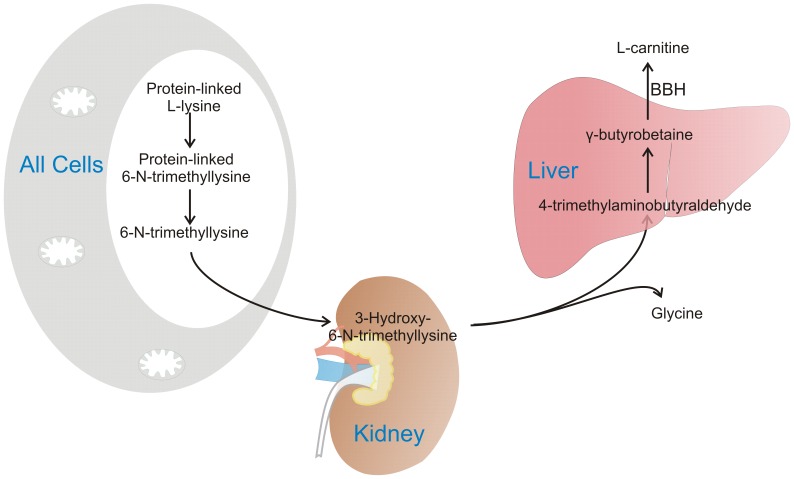
L-carnitine biosynthesis. L-carnitine can be partly provided by an omnivorous diet/fish and meat in the diet, but the required levels can also be synthesized from the amino acids L-lysine and L-methionine. L-carnitine biosynthesis takes place mainly in skeletal muscle, kidney and liver. Lysine is methylated three times by a methyltransferase that uses S-adenosyl-L-methionine as methyl group donor [42], [43]. This gives protein-linked 6-N-trimethyllysine, which is released by protein breakdown, the rate-limiting step in L-carnitine biosynthesis [44]. The major proportion of 6-N-trimethyllysine is found in skeletal muscle (65%), which quantitatively is the most important organ in L-carnitine synthesis. The next step, hydroxylation of trimethyllysine to 3-hydroxy-6-N-trimethyllysine (by 6-N-trimethyllysine hydroxylase) takes place in the mitochondria, with especially high enzyme activity in kidney but also in liver, skeletal muscle, heart and brain [45], [46]. The enzymes involved in cleavage of 3-hydroxy-6-N-trimethyllysine into glycine and 4-trimethylaminobutyraldehyde/γ-butyrobetainaldehyde (3-hydroxy-6-N-trimethyllysine aldolase), and subsequently to γ-butyrobetaine (4-trimethylaminobutyraldehyde dehydrogenase) has highest activity in the liver of rodents, and in the liver and kidney of humans. Finally, γ-butyrobetaine hydroxylase is involved in the formation of L-carnitine in the liver of rodents, and in the liver and kidney of humans [47].

L-carnitine is a water-soluble quaternary amine compound that can be synthesized from trimethylated lysine residues derived from protein degradation (Fig. 1). The synthesis takes place in liver, muscle, and kidneys, and is influenced by PPARα activation [20]. L-carnitine is mainly known to facilitate mitochondrial fatty acid import through carnitine palmitoyltransferase (CPT) I. Another important role is to maintain the mitochondrial balance between acyl-coenzyme A (CoA) and acylcarnitines, and to aid the export of surplus, potentially toxic carbon fuels in situations where catabolism of fatty acids and proteins exceed the energy requirements [21]. Acetylcarnitines of different carbon-length are end- or intermediate products of amino acid, glucose or fatty acid catabolism. Accumulation of acylcarnitines in plasma and tissues during insulin resistance is mainly caused by incomplete fatty acid oxidation and has been linked to impaired substrate switching [22], [23], [24], which could indicate dysregulation of mitochondria. Thus, acylcarnitines seem to be important both as biomarkers and mediators of metabolic disease.

The aim of the present study was to investigate whether TTA, a relatively potent PPARα ligand, and n−3 PUFAs, which are weak PPARα agonists, induced alterations of amino acid metabolism in rats fed a high fat diet. The study was performed during 50 weeks, which limited the possibility to observe first-line effects of the treatments, but could help reveal possible adverse metabolic effects after long-term treatment. In addition, plasma acylcarnitine esters were analyzed to reflect fatty acid and amino acid degradation products during long-term dietary treatment with TTA and FO. With the high dose of FO used, n−3 PUFAs will act as metabolic regulators and also be important substrates for β-oxidation. Thus, they could potentially affect energy metabolism and acylcarnitine levels through both these mechanisms.

## Materials and Methods

### Ethics Statement

This study was approved by the Norwegian Committee for Experiments on Animals (“Forsøksdyrutvalget”, permit number 2005140) and in accordance with the Norwegian legislation and regulations governing experiments using live animals. The experiments were performed in accordance with the regulations laid down by the National Animal Research Authority. The Animal Care and Use Program at University of Bergen was accredited by AAALAC international in March 2012.

### Animals and Diets

Eight to ten-weeks old male Wistar rats were obtained from Taconic Europe A/S. The animals were housed five per cage, as previously described [8]. The rats were divided into four groups and fed a 25% (w/w) high fat diet (Control) or high fat diets supplemented with TTA, FO or TTA and FO as described in Table 1. The constituents of the diets were lard (Ten Kate Vetten BV, Musselkanaal, Netherlands), soy oil, cornstarch, dyetrose, sucrose, fiber, AIN-93G mineral mix, AIN-93 vitamin mix, L-Cysteine, Choline bitartrate (Dyets Inc., Bethlehem, PA, USA), and tert-Butyl-hydroquinone (Sigma-Aldrich, St. Louis, MO, USA). TTA was synthesized as previously described [25]. FO (EPAX 6000 TG®) was the generous gift of EPAX A/S (Ålesund, Norway). The rats were anaesthetized with isofluorane (Forane, from Abbot Laboratories Ltd, IL, USA) inhalation under non-fasting conditions, 4–6 hours after the beginning of the light cycle. Blood was drawn by cardiac puncture and collected in BD Vacutainer tubes containing EDTA (Becton, Dickinson and Company, Plymouth, UK).

**Table 1:** Composition of the experimental high fat (25%, w/w) diets (g/kg diet)^1^.

Ingredients	Control	TTA	FO	TTA+FO
Lard	230	226.3	130	126.3
Soy oil	20	20	20	20
TTA (0.375%, w/w)	–	3.75	–	3.75
FO (10%, w/w)	–	–	100	100
Casein^2^	238.7	238.7	238.7	238.7
Cornstarch	397	397	397	397
Dyetrose	132	132	132	132
Sucrose	100	100	100	100
Fiber	50	50	50	50
AIN-93G mineral mix	35	35	35	35
AIN-93 vitamin mix	10	10	10	10
L-Cysteine	3	3	3	3
Choline bitartrate	2.5	2.5	2.5	2.5
TBHQ	0.014	0.014	0.014	0.014

Abbreviations: FO, fish oil; TBHQ, tertiary butylhydroquinone; TTA, tetradecylthioacetic acid.

1 The diets were isoenergetic and isonitrogenous and contained 20 g of protein per 100 g of diet.

2 Casein consisted of 83.8% protein and 0.2% fat.

### Plasma Free Amino Acid Composition

EDTA plasma from rats (200 µl) was deproteinized by adding 10% sulfosalicylic (1∶1, v/v) with Norleucine as internal standard (final 0.5 mM) and stored for 1 hour at 4°C before centrifugation at 8000 rpm for 30 min as described in Liaset et al. [26]. The supernatant was filtered and amino acid were characterised by a Biochrom 20 plus amino acid analyser as previously described [27].

### Plasma Carnitine Composition

Free L-carnitine, short-, medium-, and long-chain acylcarnitines, and the precursors for carnitine, trimethyllysine and γ-butyrobetaine, were analysed in plasma using LC-MS/MS as described previously [28] with some modifications [29].

### Gene Expression Analysis

Total cellular RNA was purified from frozen liver samples, and cDNA was produced as previously described [8]. Real-time PCR was performed with Sarstedt 384 well multiply-PCR Plates (Sarstedt Inc., Newton, NC, USA) on the following genes, using probes and primers from Applied Biosystems (Foster City, CA, USA): aminoadipate aminotransferase (*Aadat*, Rn00567882_m1); aldehyde dehydrogenase 9 family, member 1 (*Aldh9a1*, Rn01491039_m1); aldehyde oxidase 3 (*Aox3*, Rn01441420_m1); arginase (*Arg1*, Rn00691090_m1); argininosuccinate lyase (*Asl*, Rn01480437_g1); argininosuccinate synthetase (*Ass*, Rn00565808_g1); gamma-butyrobetaine hydrolase 1 (*Bbox1*, Rn00575255_m1); cystathione-beta-synthase (*Cbs*, Rn00560948_m1); ClpX caseinolytic peptidase×homolog (E.coli) (*Clpx*, Rn01418137_m1); cytochrome P450, family 7, subfamily B, polypeptide 1 (*Cyp7b1*, Rn01461859_m1); glutamate dehydrogenase 1 (*Glud1*, Rn00561306_m1); glutamate-ammonia ligase (*Glul*, Rn01483108_m1); glycerate kinase (*Glyctk*, Rn01446603_g1); histidine decarboxylase (*Hdc*, Rn00566665_m1); serine hydroxymethyltransferase (*Shmt2*, Rn01768052_g1); trimethyllysine hydroxylase, epsilon (*Tmlhe*, Rn00591314_m1). Three different reference genes were included: 18*s* (Kit-FAM-TAMRA (Reference RT-CKFT-18s)) from Eurogentec (Seraing, Belgium), glyceraldehyde-3-phosphate dehydrogenase (*Gapdh*, Mm99999915_g1) from Applied Biosystems, and ribosomal protein, large, P0 (*Rplp0*, Gene ID 11837) from Thermo Fisher Scientific Inc. (Waltham, MA, USA). To normalize the absolute quantification according to the reference genes, a second set of PCR was performed for all experimental samples. The relative abundance values were calculated for the reference genes as well as for the target genes using standard curves derived from Universal Rat Reference RNA (Agilent Technologies Inc., Santa Clara, CA, USA). The NormFinder software was used to evaluate the reference genes [30], and data normalized to *18s* are presented.

### Statistical Analyses and Presentation of Data

The results were presented as means with their standard deviations (SD) of 10 rats per group. Gene expression data was normalized against the control diet group. Data were evaluated by two-way ANOVA for treatment additivity and synergy, and p-values <0.05 were considered significant [31]. Results were not adjusted for multiple comparisons. Statistics were performed using PASW Statistics for Windows, version 18 (SPSS Inc., Chicago, IL, USA).

## Results

### Basic Characteristic of the Feeding Groups

In this 50-week long-term study all feeding groups had the same average feed intake (Table 2). However, the diets supplemented with TTA or a combination of TTA and FO led to a reduction in body weight gain, while FO fed rats demonstrated identical weight gain to control high-fat fed rats. The liver weights of the rats were increased in the TTA groups, as were the hepatic indexes.

**Table 2:** Weight and feed data in rats on 50 week of diet administration.

	Dietary supplementation^1^				Statistical significance of variance ratio (P)^2^, effects of		
	Control	TTA	FO	TTA+FO	TTA	FO	TTA*FO
**Start weight**	262±33.3	268±29.3	260±24.7	254±14.0	0.93	0.23	0.26
**End weight**	577±104	508±106	571±87.0	484±58.5	0.001	0.48	0.69
**Weight gain**	316±90.2	240±99.1	310±82.4	229±55.3	<0.001	0.66	0.90
**Liver weight**	12.4±2.4	22.8±6.1	12.6±1.8	15.8±1.6	<0.001	<0.001	<0.001
**Hepatic index** 3	21.6±2.2	45.4±6.7	22.5±2.2	33.8±3.6	<0.001	<0.001	<0.001
**Feed intake** 4	15.1	15.7	14.9	15.7			
**Feed efficiency** 5	0.060±0.017	0.044±0.018	0.059±0.016	0.042±0.010	<0.001	0.75	0.85

Abbreviations: TTA, tetradecylthioacetic acid; FO, fish oil.

1 Values are mean ± SD (n = 10).

2 P-values from two-way ANOVA, where TTA*FO denotes the interaction effect.

3 Ratio of whole liver (g) to body weight (kg).

4 Values in g per rat per day.

5 Ratio of weight gain (g) to feed intake (g).

**Figure 2:**
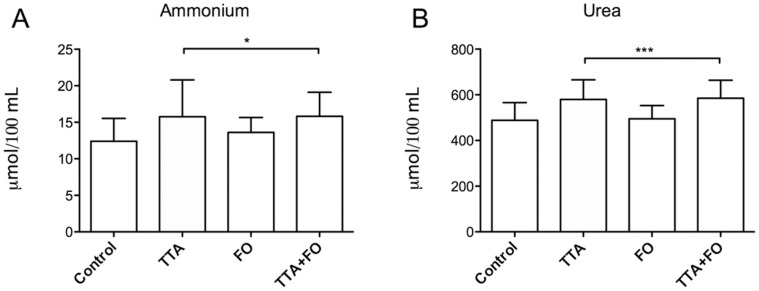
Plasma levels of ammonium and urea increased with TTA-treatment. Male Wistar rats were fed high fat diets supplemented with 0.375% (w/w) tetradecylthioacetic acid (TTA), 10% (w/w) fish oil (FO) or a combination of TTA and FO (TTA+FO) for 50 weeks, and ammonium (A) and urea (B) was measured. Values given are means ± SD (n = 10). Asterix indicates statistical significance of variance ratio and effects of TTA analyzed by two-way ANOVA, and clams indicate the two diet groups that contain TTA (*p<0.05; ***p<0.001).

By-products of liver amino acid catabolism were investigated in plasma at the end of the 50-week feeding period. The TTA diet, but not the FO diet, significantly increased the plasma concentrations of ammonia and urea, compared to the control high fat diet (Fig. 2 and Table S1).

### Plasma Amino Acids and Metabolites

The plasma concentrations of the following glucogenic free amino acids were increased by TTA compared to control: asparagine, aspartic acid, glutamine, glycine, histidine, serine, valine (Table 3). The TTA diet also increased the plasma levels of phenylalanine, threonine, thryptophane, and tyrosine, which are both ketogenic and glucogenic, while the ketogenic amino acids leucine and isoleucine only showed a small but still significant increase. The important bile-component taurine showed a small, but significant increase with TTA and TTA+FO. The α-amino acid citrulline and another important product in the urea cycle, ornithine, was increased by TTA compared to control. Thus, both essential and non-essential amino acids increased with the TTA diet, in addition to an increase in products of the urea cycle. Moreover, arginine, lysine, and 1-methylhistidine were the only amino acids present in significantly lower concentrations in TTA administrated animals compared to control. 1-methyl-histidine was also significantly reduced in the TTA group, but not in the TTA+FO group. In contrast, FO only changed the composition of a few amino acids compared to control and the following elevated amino acids and amino acid derivatives were: asparagine, arginine, glutamate, serine, and 1-methyl-histidine.

**Table 3:** Plasma levels of free amino acids and amino acid derivatives (µmol/100 mL) in rats after 50 weeks of diet administration.

	Dietary supplementation^1^				Statistical significance of variance ratio (P)^2^, effects of		
	Control	TTA	FO	TTA+FO	TTA	FO	TTA*FO
**Asparagine**	9.85±2.03	14.85±2.69	12.87±3.99	16.89±2.01	<0.001	0.007	0.58
**Arginine**	8.87±1.57	5.60±1.37	10.29±2.51	8.30±2.10	<0.001	0.002	0.30
**Aspartic acid**	1.37±0.28	2.00±0.40	1.70±0.31	1.92±0.53	0.002	0.32	0.12
**Citrulline**	7.35±1.51	12.00±2.96	7.98±2.35	9.60±1.86	<0.001	0.22	0.04
**Glutamine**	53.85±5.04	71.64±13.26	60.11±9.97	71.30±8.88	<0.001	0.34	0.29
**Glutamate**	10.82±3.15	14.83±2.63	16.36±2.94	15.41±3.37	0.12	0.003	0.01
**Glycine**	23.44±4.87	37.46±11.88	25.08±3.28	40.11±11.65	<0.001	0.45	0.86
**Histidine**	5.93±0.80	15.67±6.99	8.06±1.66	11.39±1.53	<0.001	0.36	0.01
**1-Methylhistidine**	1.01±0.50	0.76±0.37	1.61±0.49	0.99±0.48	0.006	0.008	0.20
**Phenylalanine**	5.60±0.83	7.95±1.17	7.09±1.13	7.15±0.85	0.001	0.30	0.001
**Serine**	34.96±8.01	59.80±13.53	43.27±5.30	72.18±14.00	<0.001	0.005	0.56
**Taurine**	18.05±2.95	20.88±4.11	16.12±1.75	25.61±8.12	<0.001	0.37	0.04
**Threonine**	41.89±10.75	91.64±28.64	26.66±7.23	90.11±17.77	<0.001	0.15	0.24
**Tryptophane**	119±31.21	197±63.70	134±17.41	173±38.02	<0.001	0.74	0.14
**Tyrosine**	9.10±1.77	15.72±3.75	12.01±3.49	12.36±2.57	0.001	0.82	0.002
**Valine**	18.29±3.26	22.68±4.32	17.70±2.93	22.81±2.57	<0.001	0.83	0.73
**Alanine**	61.68±14.24	74.89±13.15	73.20±21.45	66.58±10.26	0.50	0.74	0.05
**Cysteine**	0.41±0.45	0.52±0.73	0.47±0.92	0.87±0.74	0.27	0.38	0.51
**Isoleucine**	7.20±1.34	7.86±1.58	6.53±1.30	7.69±1.27	0.04	0.34	0.57
**Leucine**	13.48±2.51	15.21±3.40	13.76±2.26	15.94±2.47	0.03	0.55	0.79
**Lysine**	53.16±6.03	45.19±11.06	54.60±12.19	46.67±6.26	0.01	0.62	0.99
**Methionine**	6.24±0.86	6.64±0.77	6.94±1.29	6.87±0.98	0.60	0.14	0.46
**Ornithine**	5.74±1.94	7.65±2.02	6.14±0.90	7.30±1.57	0.006	0.97	0.48
**Phenylethylamine**	7.43±1.27	6.72±1.24	6.21±1.56	7.59±1.01	0.42	0.67	0.02
**Phosphatidylserine**	2.80±0.34	3.09±0.78	3.03±0.51	3.03±0.38	0.42	0.61	0.40
**Proline**	32.69±7.51	36.18±10.47	36.77±11.35	31.14±8.55	0.50	0.86	0.08

1 Values are mean ± SD (n = 10).

2 P-values from two-way ANOVA, where TTA*FO denotes the interaction effect.

We looked at the amino acids influenced by both treatments, to determine whether TTA and FO affected the outcome independently of the each other (no interaction effect by TTA*FO, p>0.05). Additive effects of TTA and FO were observed on the plasma levels of asparagine and serine, and opposing effects were observed on arginine and 1-methylhistidine levels (Table 3).

We studied the expression of selected hepatic genes involved in amino acid metabolism, and found that genes involved in amino acid catabolism were either unchanged (*Cbs*), or increased in expression (*Shmt2*, *Glyctk*, *Glud1*) by TTA-treatment (Table 4). *Aadat*, a gene from the saccaropine pathway, a major pathway for L-lysine catabolism, demonstrated increased hepatic expression after TTA treatment, simultaneous with decreased lysine levels. The hepatic expression of *Glul*, involved in glutamine formation, was unchanged by TTA and FO. *Hdc*, involved in the formation of histamine from hisitine, was highly increased by both TTA and FO-treatment. Other genes involved in amino acid metabolism, previously showed to be upregulated with FO [32], tended to be upregulated (*Clpx*), or showed no change (*Cyp7b1, Aadat*, *Aox3*) by FO in this study. We also observed a specific decrease in *Arg1* by FO. The expression of *Ass1* and *Asl*, genes involved in the urea cycle, was reduced by TTA.

**Table 4:** Gene expression of selected genes in liver of rats after 50 weeks of diet administration^1^.

	Dietary supplementation^2^				Statistical significance of variance ratio (P)^3^, effects of		
	Control	TTA	FO	TTA+FO	TTA	FO	TTA*FO
***Aldh9a1***	1.00±0.20	1.31±0.18	1.02±0.61	1.11±0.16	0.07	0.40	0.31
***Bbox1***	1.00±0.27	1.13±0.21	0.86±0.27	1.05±0.26	0.05	0.18	0.65
***Tmlhe***	1.00±0.22	1.15±0.13	0.98±0.26	1.02±0.13	0.15	0.26	0.34
***Aox3***	1.00±0.22	0.31±0.16	0.83±0.47	0.33±0.15	<0.001	0.38	0.30
***Clpx***	1.00±0.33	2.76±0.87	1.48±0.42	2.81±0.73	<0.001	0.20	0.29
***Cyp7b1***	1.00±0.31	0.97±0.34	0.93±0.34	0.69±0.14	0.17	0.11	0.28
***Aadat***	1.00±0.20	1.41±0.23	0.96±0.25	1.45±0.36	<0.001	0.98	0.65
***Glyctk***	1.00±0.19	1.21±0.19	0.98±0.22	1.04±0.11	0.03	0.10	0.17
***Cbs***	1.00±0.31	1.03±0.38	0.78±0.24	1.16±0.21	0.03	0.66	0.07
***Shmt2***	1.00±0.31	1.31±0.38	0.84±0.22	1.19±0.44	0.005	0.19	0.85
***Hdc***	1.00±0.70	57.83±20.87	10.43±3.91	71.74±30.43	<0.001	0.05	0.69
***Glul***	1.00±0.43	1.03±0.34	0.90±0.26	1.12±0.38	0.28	0.93	0.38
***Glud1***	1.00±0.27	1.57±0.28	0.94±0.37	1.40±0.34	<0.001	0.24	0.60
***Arg1***	1.00±0.38	0.86±0.23	0.58±0.17	0.69±0.23	0.88	0.001	0.13
***Ass1***	1.00±0.43	0.41±0.19	0.57±0.39	0.44±0.16	0.001	0.06	0.03
***Asl***	1.00±0.28	0.59±0.15	0.78±0.24	0.61±0.10	<0.001	0.11	0.07

Abbreviations: TTA, tetradecylthioacetic acid; FO, fish oil; *Aldh9a1*, aldehyde dehydrogenase 9, subfamily A1; *Bbox1*, gamma-butyrobetaine hydroxylase 1; *Tmlhe*, trimethyllysine hydroxylase, epsilon; *Aox3*, aldehyde oxidase 3; *Clpx*, ClpX caseinolytic peptidase×homolog; *Cyp7b1*, cytochrome P450, family 7, subfamily B, polypeptide 1; *Aadat*, aminoadipate aminotransferase; *Glyctk*, glycerate kinase; *Cbs*, cystathionine beta synthase; *Shmt2*, serine hydroxymethyltransferase; *Hdc*, histidine decarboxylase; *Glul*, glutamate-ammonia ligase; *Glud1*, glutamate dehydrogenase 1; *Arg1*, arginase, liver; *Ass1*, argininosuccinate synthase 1; *Asl*, argininosuccinate lyase.

1 Relative to control diet.

2 Values are mean ± SD (n = 10).

3 P-values from two-way ANOVA.

### Plasma L-carnitine, Carnitine Precursors, and Acylcarnitines

**Figure 3:**
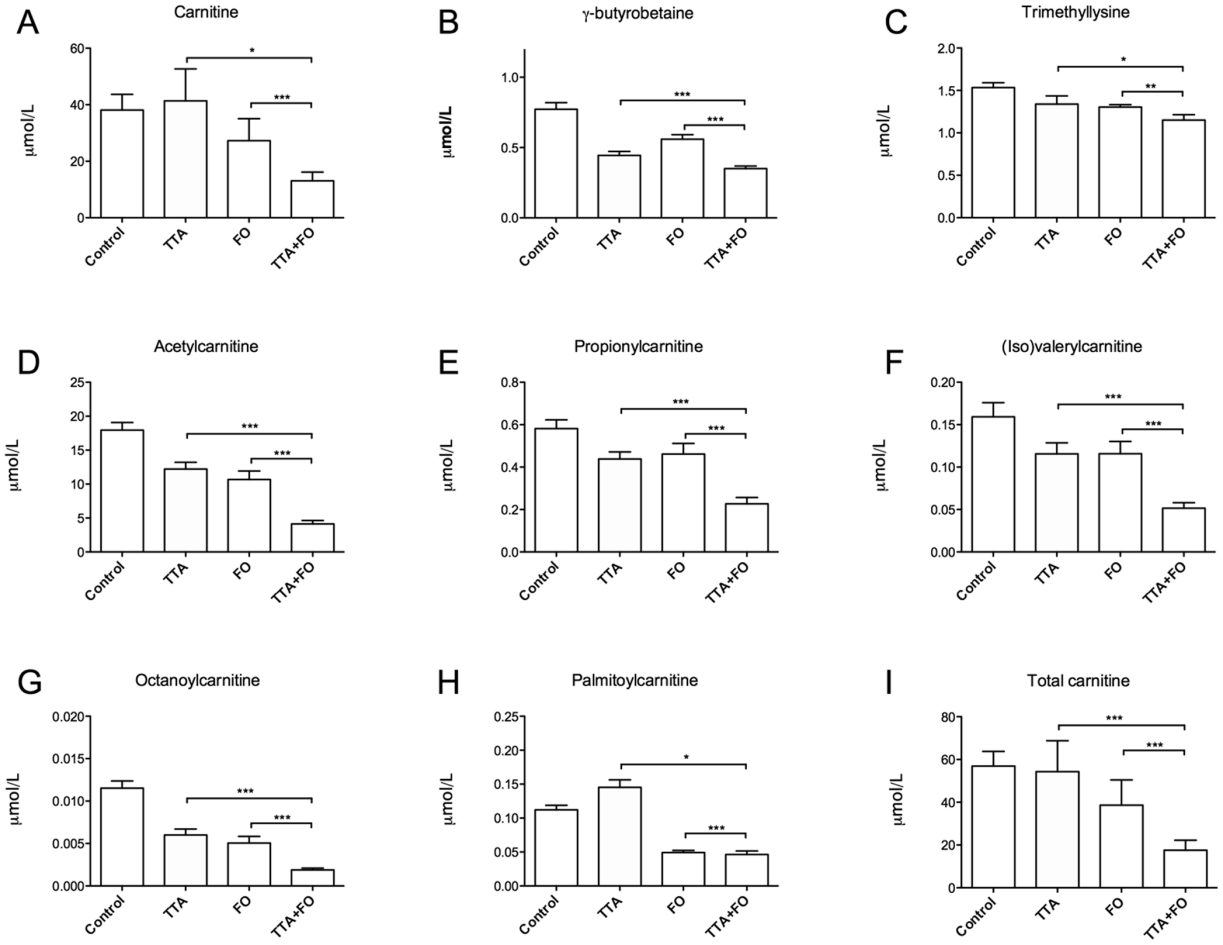
Plasma levels of L-carnitine and acylcarnitines were affected by both TTA and fish oil. Male Wistar rats were fed high fat diets supplemented with 0.375% (w/w) tetradecylthioacetic acid (TTA), 10% (w/w) fish oil (FO) or a combination of TTA and FO (TTA+FO) for 50 weeks, and L-carnitine (A), L-carnitine precursors (B-C) and acylcarnitines (D-H) were measured in plasma. Values given are means ± SD (n = 10). Asterix indicates statistical significance of variance ratio and effects of TTA (long clamps) and FO (short clamps) analyzed by two-way ANOVA (*p<0.05; **p<0.01; ***p<0.001).

We found that plasma free L-carnitine concentrations were reduced by FO treatment but unchanged by TTA treatment (Fig. 3A). The carnitine precursors γ- butyrobetaine and trimethyllysine were reduced by both treatments (Fig. 3B and C). Gene expression analysis demonstrated a TTA-induced increase in the important genes in L-carnitine biosynthesis, *Aldh9a1*, *Bbox1* and *Tmlhe*, however only reaching significance for *Bbox1* (Table 4).

Plasma acetylcarnitine, octanoylcarnitine, propionylcarnitine, and valeryl−/isovalerylcarnitine were reduced in by both TTA- and FO diets compared to the control high-fat diet (Fig. 3D–G). FO also reduced the concentration of palmitoylcarnitine, while TTA treatment gave a small but significant increase on this parameter (Fig. 3H). The co-treatment with TTA and FO reduced plasma levels of acetylcarnitine, octanoylcarnitine, propionylcarnitine and valeryl−/isovalerylcarnitine compared to TTA or FO alone. The p-values for interaction effects of TTA and FO treatment on these acylcarnitines are given in Table S1. The lack of an interaction effect indicated that TTA and FO affected these parameters additively.

While the total plasma L-carnitine level, calculated as the sum of free carnitine and carnitine esters, was unchanged in the control group versus the TTA group, this number was reduced in the FO diet groups compared to control (Fig. 3I). Two-way ANOVA indicated a synergistic reduction of total carnitine and free L-carnitine by TTA and FO (Table S1).

## Discussion

In this 50 weeks study in rats fed a high fat diet (25%, w/w) we demonstrated that the pan-PPAR agonist TTA increased plasma levels of several essential and non-essential amino acids, simultaneous with reduced body weight gain. We previously reported a reduction in plasma lipids linked to increased hepatic β-oxidation in these animals [8]. This may suggest that treatment with TTA, which has its main effect on PPARα, is associated with energy transformation in which surplus energy from fat is transferred to amino acids. FO on the other hand had a moderate effect on plasma amino acid levels and no effect on body weight, despite the previously reported increased hepatic β-oxidation, reduced lipogenesis, and similar TAG-lowering as TTA [8]. Notably, combined TTA and FO treatment additively lowered plasma TAG levels [8], and acylcarnitine levels, and resulted in a 70% reduction in total plasma carnitine. In contrast, the TTA and FO combination diet had less effect on amino acid levels than TTA alone.

An increase in most glucogenic amino acids were observed upon TTA feeding. This could indicate a reduced entry of amino acids into the Krebs cycle for ATP production, and thus conservation of glucogenic amino acids in a situation with an ample amount of energy derived from fatty acids. Long-term TTA treatment showed a similar effect as 4 weeks WY 14,643 treatment on all amino acids, including a specific reduction of arginine, supporting a PPARα-induction effect. The exceptions were asparagine, which was only upregulated by TTA, and lysine, which was upregulated by WY 14,643 and downregulated by TTA [4], [5].

The general increase in both essential and non-essential amino acids could be consistent with TTA-treatment-induced increase in protein turnover. PPARα-agonists are reported to induce hepatic hypertrophy, and proliferation of mitochondria and peroxisomes [33], [34], [35], and these pleiotropic effects could possibly give rise to increased recruitment of amino acids to the liver. We observed a striking increase in liver weight by 83.8% in the animals on TTA-diet compared to control diet after 50 weeks. Deamination of amino acids releases ammonia, which can efficiently be converted to urea by the urea cycle. The increase of both urea and ammonium in plasma with TTA could indicate higher amino acid turnover. Moreover, citruline and ornithine were increased by TTA alone and in combination with FO. Sheikh et al. [5] suggested that similar findings with WY 14,643 could be due to larger activity of the hypertrophic liver. In contrast, studies in PPARα-deficient animals demonstrated an increased urea production in fasted animals, indicating an inhibitory effect of PPARα on the urea cycle [5]. Our findings demonstrate that while the products of the urea cycle were increased, the gene expression of key enzymes was downregulated, indicating that feedback mechanisms could be involved during long term PPARα activation.

A previous study demonstrated increased amino acid catabolism in mice lacking PPARα [36]. Thus, we investigated whether PPARα-activation induced a decrease in amino acid catabolism. However, the selected hepatic genes were either unchanged (*Cbs*) or increased in expression (*Glyctk*, *Glud1*, *Hdc*) with TTA-treatment. This is in contrast to short-term experiments with PPARα agonists, where downregulation of amino acid catabolic genes was observed [5], [32]. In a proteomic analysis of liver mitochondria from the same animals as the current study, proteins involved in amino acid catabolism were mainly downregulated [37]. The increase in expression of some genes could be a long-term compensatory regulatory mechanism to an increased input of amino acids to the liver, not reflected at the protein level.

The hepatic expression of selected genes involved in the synthesis of amino acids was not affected by either treatment. *Glul*, involved in glutamine formation, was unchanged, while plasma glutamine increased with TTA. And similarly, TTA and FO additively increased the serine level, but serine hydroxymethyltransferase 2 expression was unchanged by either treatment. In total, the TTA- or FO-induced increase in plasma amino acid levels did not seem to be caused by increased synthesis of amino acids in the liver.

FO and TTA had mostly non-overlapping effects on plasma amino acids. FO feeding increased arginine and glutamate, which were reduced or unchanged by TTA, respectively. Asparagine and serine were increased by both treatments, but as additive effects were observed in the combination group, different mechanisms may be involved. Thus, although some amino acids were increased by FO treatment, the major effect of FO seemed to be plasma lipid lowering. Since FO activates PPARα, as seen by increased expression of PPARα-regulated genes in a previous publication from this study [8], we could expect similar effects by FO and TTA on plasma amino acids. When this was not the case, FO may have induced other mechanisms opposing the PPARα-effect. A genomic study comparing fenofibrate and FO demonstrated a specific upregulation of genes involved in amino acid metabolism with FO-treatment [32]. Some of these genes were also upregulated by FO in our experiment, however not significantly. A proteomics analysis of liver mitochondria from this study demonstrated opposite regulation of a number of proteins involved in amino acid metabolism by FO and TTA [37]. Thus, it is possible that FO activates mechanisms that counteract the PPARα effect on plasma amino acids.

L-carnitine is a conditionally essential nutrient necessary for mitochondrial oxidation, through the import and export of acyl-CoA. We recently showed that two weeks supplementation of TNFα-transgenic mice with TTA or n−3 PUFAs resulted in increased plasma levels of acetyl- and propionylcarnitine, demonstrating an increase in β-oxidation- and amino acid degradation end products during short-term treatments [38], [39]. Thus, the reduction in short-chained acylcarnitines after long-term treatment with TTA or FO may indicate that the oxidative system has adapted to the increased fatty acid oxidation level, and is able to efficiently utilize the short-chained acylcarnitines. Moreover, all diets suggest a complete fatty acid oxidation of medium-chain fatty acids. In contrast, TTA alone increased the plasma concentration of 16C palmitoylcarnitine, perhaps reflecting the presence of slowly degradable TTA-carnitine. However, the levels were not high enough to be reflected in plasma non-esterified fatty acids (reported previously, [8]). The additive effects of combined TTA- and FO-treatment on the reduction of acylcarnitine esters agrees with previous findings that TTA and FO lowers plasma lipids through partly different mechanisms [8]. One possible explanation to this additive reduction could be reduced substrate availability for β-oxidation due to the depletion of adipose tissue and lowering of plasma lipids in rats co-treated with TTA and FO for 50 weeks.

It was of interest that plasma γ-butyrobetaine levels were decreased by TTA administration whereas the L-carnitine level remained constant. This may due to increased consumption of γ-butyrobetaine in response to the increased demand of free L-carnitine (Fig. 3). The amino acid precursor to L-carnitine, lysine, was also reduced in the plasma of rats on TTA-diets. As previously mentioned, this is in contrast to a short-term study with a PPARα agonist, where lysine was increased [5]. Thus, plasma lysine reduction could be an effect of long-term treatment with a hypolipidemic agent increasing the demand for carnitine. Genes regulating L-carnitine biosynthesis are activated during fasting and by PPARα ligands [20], [40]. PPARα deficient mice demonstrate reduced expression of genes involved in L-carnitine biosynthesis and transport, as well as reduced L-carnitine levels [36]. We observed an increase in the expression of *Bbox1* and *Aldh9a1,* genes important for L-carnitine biosynthesis, by TTA. Thus, PPARα activation of L-carnitine biosynthesis in TTA-treated animals may have maintained the plasma L-carnitine level compared to control. In contrast, FO and in particular FO+TTA led to a significant reduction in free L-carnitine and a reduced total carnitine level, which could indicate sequestration of carnitine in the tissues. This effect of FO on carnitine homeostasis could have important effects on mitochondrial function [21], and should be further studied. It is possible that the substantial amount of n−3 PUFAs in the FO-diets influenced the plasma carnitine level through their role as substrates for β-oxidation. The double bonds in n−3 PUFAs have to be moved in order for β-oxidation to proceed, and this requires the activity of the specific enzymes enoyl-CoA isomerase 1 and 2, and 2,4-dienoyl-CoA-reductase 1 and 2 [41]. This may potentially slow down the β-oxidation process and alter the balance between acyl-CoA and acylcarnitines. A method for the analysis of L-carnitine and acylcarnitines in liver, heart, and muscle was unfortunately not available.

In conclusion, in addition to its known TAG-lowering effect, TTA regulates the metabolism of amino acids and L-carnitine biosynthesis, most probably via activation of PPARα, either through direct regulation of gene expression or through indirect mechanisms. FO had a major effect on plasma acylcarnitines, similar to TTA, and specifically reduced L-carnitine levels, but had a modest effect on amino acid levels. This may be due to regulation of genes involved in amino acid metabolism through alternative pathways, and a lower level of PPARα activation. Our results indicate that the previously reported increased fatty acid oxidation and weight loss in male Wistar rats treated with TTA is accompanied by reduced catabolism of amino acids, and thus may reflect energy transformation from lipids to amino acids. The possible impact on health of the reduced plasma L-carnitine levels by FO, and the alterations in amino acid metabolism by specific PPARα-agonists require further studies.

## Supporting Information

Table S1 **Plasma levels of ammonium, urea, L-carnitine and its precursors, and acylcarnitines in rats after 50 weeks of diet administration.** Mean values ± SD (n = 10), and the statistical significance of variance ratio (P)^2^, effects of tetradecylthioacetic acid (TTA), fish oil (FO) and TTA*FO from two-way ANOVA, is given in this table.
(DOCX)
