# Supplementary material for: Fish Oil and the Pan-PPAR Agonist Tetradecylthioacetic Acid Affect the Amino Acid and Carnitine Metabolism in Rats
Source: PLoS One. 2013 Jun 24;8(6):e66926. doi: 10.1371/journal.pone.0066926 (PMC3691320; doi:10.1371/journal.pone.0066926)
Supplement: Table S1 — Plasma levels of ammonium, urea, L-carnitine and its precursors, and acylcarnitines in rats after 50 weeks of diet administration. Mean values ± SD (n = 10), and the statistical significance of variance ratio (P)2, effects of tetradecylthioacetic acid (TTA), fish oil (FO) and TTA*FO from two-way ANOVA, is given in this table. (DOCX) [file pone.0066926.s001.docx]

**Table S1.** Plasma levels of ammonium, urea, L-carnitine and its precursors, and acylcarnitines in rats after 50 weeks of diet administration

|  | **Dietary supplementation^1^** | | | |  | **Statistical significance of**  **variance ratio (P)^2^, effects of** | | |
| --- | --- | --- | --- | --- | --- | --- | --- | --- |
|  | **Control** | **TTA** | **FO** | **TTA + FO** |  | **TTA** | **FO** | **TTA*FO** |
| **Ammonium (µmol/100mL)** | 12.41 ± 3.12 | 15.76 ± 5.03 | 13.61 ± 2.05 | 15.82 ± 3.29 |  | 0.02 | 0.58 | 0.61 |
| **Urea (µmol/100mL)** | 488 ± 77.69 | 580 ± 85.86 | 495 ± 57.83 | 585 ± 78.43 |  | 0.001 | 0.80 | 0.97 |
| **Trimethyllysine (µmol/L)** | 1.53 ± 0.18 | 1.34 ± 0.30 | 1.30 ± 0.09 | 1.15 ± 0.20 |  | 0.01 | 0.003 | 0.75 |
| **γ-Butyrobetaine (µmol/L)** | 0.77 ± 0.15 | 0.44 ± 0.09 | 0.56 ± 0.10 | 0.35 ± 0.06 |  | <0.001 | <0.001 | 0.08 |
| **Free L-carnitine (µmol/L)** | 38.13 ± 5.53 | 41.40 ± 11.28 | 27.26 ± 7.77 | 13.09 ± 3.07 |  | 0.03 | <0.001 | 0.001 |
| **Acetylcarnitine (µmol/L)** | 17.96 ± 3.60 | 12.21 ± 3.15 | 10.68 ± 3.96 | 4.13 ± 1.61 |  | <0.001 | <0.001 | 0.70 |
| **Octanoylcarnitine (µmol/L)** | 11.5 ± 2.7^3^ | 6.0 ± 2.2^3^ | 5.1 ± 2.5^3^ | 1.9 ± 0.7^3^ |  | <0.001 | <0.001 | 0.10 |
| **Palmitoylcarnitine (µmol/L)** | 0.11 ± 0.02 | 0.15 ± 0.04 | 0.05 ± 0.01 | 0.05 ± 0.02 |  | 0.04 | <0.001 | 0.02 |
| **Propionylcarnitine (µmol/L)** | 0.58 ± 0.13 | 0.44 ± 0.11 | 0.46 ± 0.16 | 0.23 ± 0.10 |  | <0.001 | <0.001 | 0.26 |
| **(Iso)valerylcarnitine (µmol/L)** | 0.16 ± 0.05 | 0.12 ± 0.04 | 0.12 ± 0.05 | 0.05 ± 0.02 |  | <0.001 | <0.001 | 0.45 |
| **Total carnitine (µmol/L)** | 57.3 ± 7.1 | 54.3 ± 14.4 | 38.6 ± 11.8 | 17.5 ± 4.7 |  | 0.001 | <0.001 | 0.007 |

Abbreviations: TTA, tetradecylthioacetic acid; FO, fish oil.

^1^ Values are mean ± SD (n=10). ^2^ P-values from two-way ANOVA, where TTA*FO denotes the interaction effect. ^3^ Values are * 10^-3^.
